# Inhibition of foot-and-mouth disease virus replication *in vitro* and *in vivo* by small interfering RNA

**DOI:** 10.1186/1743-422X-5-86

**Published:** 2008-07-25

**Authors:** Wang Pengyan, Ren Yan, Guo Zhiru, Chen Chuangfu

**Affiliations:** 1 College of Animal Science and Technology, Shihezi University, Shihezi, Xinjiang, 832003, PR China

## Abstract

By using bioinformatics computer programs, all foot-and-mouth disease virus (FMDV) genome sequences in public-domain databases were analyzed. Based on the results of homology analysis, 2 specific small interfering RNA (siRNA) targeting homogenous 3D and 2B1 regions of 7 serotypes of FMDV were prepared and 2 siRNA-expression vectors, pSi-FMD2 and pSi-FMD3, were constructed. The siRNA-expressing vectors were used to test the ability of siRNAs to inhibit virus replication in baby hamster kidney (BHK-21) cells and suckling mice, a commonly used small animal model. The results demonstrated that transfection of BHK-21 cells with siRNA-expressing plasmids significantly weakened the cytopathic effect (CPE). Moreover, BHK-21 cells transiently transfected with short hairpin RNA (shRNA)-expressing plasmids were specifically resistant to the infection of the FMDV serotypes A, O, and Asia I and this the antiviral effects persisted for almost 48 hours. We measured the viral titers, the 50% tissue culture infective dose (TCID_50_) in cells transfected with anti-FMDV siRNAs was found to be lower than that of the control cells. Furthermore, subcutaneous injection of siRNA-expressing plasmids in the neck of the suckling mice made them less susceptible to infection with O, and Asia I serotypes of FMDV.

## Findings

Foot-and-mouth disease (FMD) is an acute and highly contagious disease requiring expensive treatment occurring in cloven-hoofed animals. The etiological agent of FMD is foot-and-mouth disease virus (FMDV), which belongs to the genus *Aphthovirus *of the family *Picornaviridae *[1]. The spreading capacity of the virus and its ability to change its antigenic identity make it a serious threat to the beef and dairy industries in many countries. FMDV has 7 serotypes and over 70 subtypes. Owing to the absence of reciprocal protection among all the serotypes, it is difficult to control FMD through vaccination and impossible to eliminate FMD by conservative natural breeding. A recent occurrence of a large epidemiogenesis has made the development of emergency antiviral strategies essential for preventing outbreaks of FMD.

RNA interference (RNAi) is a process of sequence-specific, posttranscriptional gene silencing (PTGS) in animals and plants, which can be induced by 21- to 23-nucleotide (nt) siRNA that demonstrates sequence homology to the target gene [2,3]. It is well known that one obvious potential function for the RNAi machinery would be to defend cells against viruses that express dsRNA as part of their life cycle [4]. Indeed, there is compelling evidence indicating that RNAi is critical incurtailing viral infections in both plants and invertebrates. Moreover, it can be readily demonstrated that the artificial induction of an antiviral RNAi response in mammalian cells can confer strong protection against a wide range of pathogenic viruses [5]. Nevertheless, it remains unclear whether RNAi is involved in antiviral defense in mammalian cells in physiological conditions. Mammalian cells were originally thought to be unlikely to posses an active RNA-silencing machinery [6], besides a nonspecific, interferon mediated antiviral response mediated by dsRNA [7,8], especially by viral long (35-nt) dsRNA [9]. The recent description of RNAi in mammalian cells proved that the RNA silencing machinery is conserved in mammals [10]. In some cases, a strong antiviral effect of RNAi was observed in the cases of human immunodeficiency virus [11,12], hepatitis B virus [13,14] and poliovirus and human papillomavirus [15,16]. In fact, several viruses have now been shown either to express their own miRNAs in infected cells or to take advantage of host cell miRNAs to enhance their replication [17-19]. It therefore seems reasonable to propose that the extremely potent interferon system has displaced RNAi as the key defense against virus infection in mammalian cells [20]. SiRNA probably operates at multiple levels in mammals, its main action is expected to be mediated at the posttranscriptional level by rapid destruction of homologous mRNAs. The use of siRNA as an antiviral agent could lead to a selective pressure on the siRNA target sequences that might result in the appearance of escape variants due to the changes in the target sequence. Thus, the selected virus target sequences were located in the conserved regions of the virus genome [21]. In this study, we describe the use of RNAi in inhibiting virus replication in BHK-21 cells and suckling mice. The selected siRNA targets had 100% identity when compared with all the FMDV sequences deposited in GenBank, regardless of their serotype. This level of identity is an indication of a strong selective pressure against mutations since this sequence resists changes during the evolution of the virus. This selective pressure could maintain the siRNA target sequences without alterations, ensuring the effective activity of the siRNAs described in the present study. This work offers an insight into the use of RNAi in animal breeding for disease resistance.

The commercial plasmid pSilencer5. 1-H1 was used to express the inverted-repeat RNA corresponding to homogenous 3D and 2B1 coding regions of the 7 serotypes of FMDV. 2 siRNAs template primers were

### FMDV-2

(p1): 5'-GATCCGCTACAGATCACCATACCTTTCAAGAGAAGGTATGGTGATCTGTAGCTTTTTTGGAAA-3'(p2): 5'-AGCTTTTCCAAAAAAGCTACAGATCACCATACCTTCTCTTGAA AGGTATGGTGATCTGTAGCG-3'

### FMDV-3

(p1): 5'-GATCCGCCAGATGCAGAGGGACATGTTCAAGAGACATGTCCCTCTGCATCTGGTTTTTTGGAAA-3'

(p2): 5'-AGCTTTTCCAAAAAACCAGATGCAGAGGGACATGTCTCTTGAACATGTCCCTCTGCATCTGGCG-3'

First, The 2 pairs of primers were annealed and ligated with the linear retrovirus vector pSilencer5. 1-H1 to produce 2 siRNA-expression vectors – pSi-FMD2 and pSi-FMD3. Sequencing confirmed the correct ligation of the two plasmids. The primer used for sequencing was: 5'-TTGTACACCCTAAG CCTCCG-3'.

**Figure 1:**
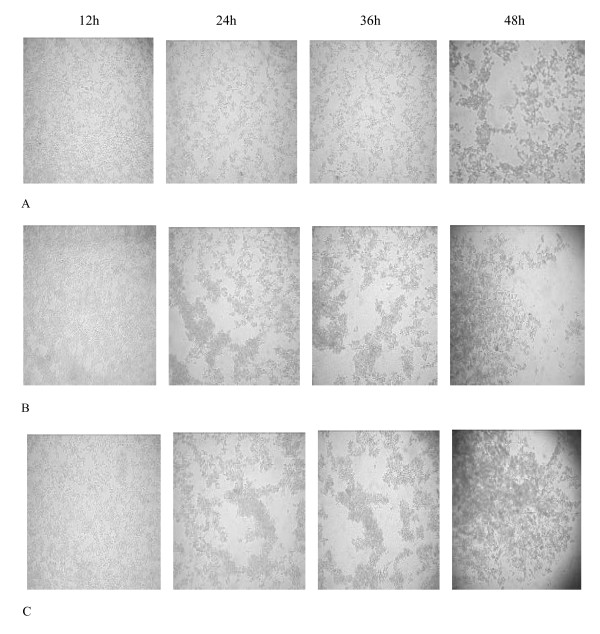
**CPEs of BHK-21 cells infected with FMDV at different times**. A. CPEs of BHK-21 cells transfected with FMDV-specific siRNA-expressing plasmid;. B. CPEs of BHK-21 cells transfected with control plasmid;. C. CPEs of Control BHK-21 cells. As showed in Fig. 1, CPEs appeared in the BHK-21 cells infected with FMDV serotype A at 12 h postinfection and were particularly severe among the 4 groups between 24 h to 36 h. Cellular detachment, rounding, and destruction of the control group were more severe than the experimental group. At 48 h postinfection, the cells of the control group were dead and almost detached.

**Figure 2:**
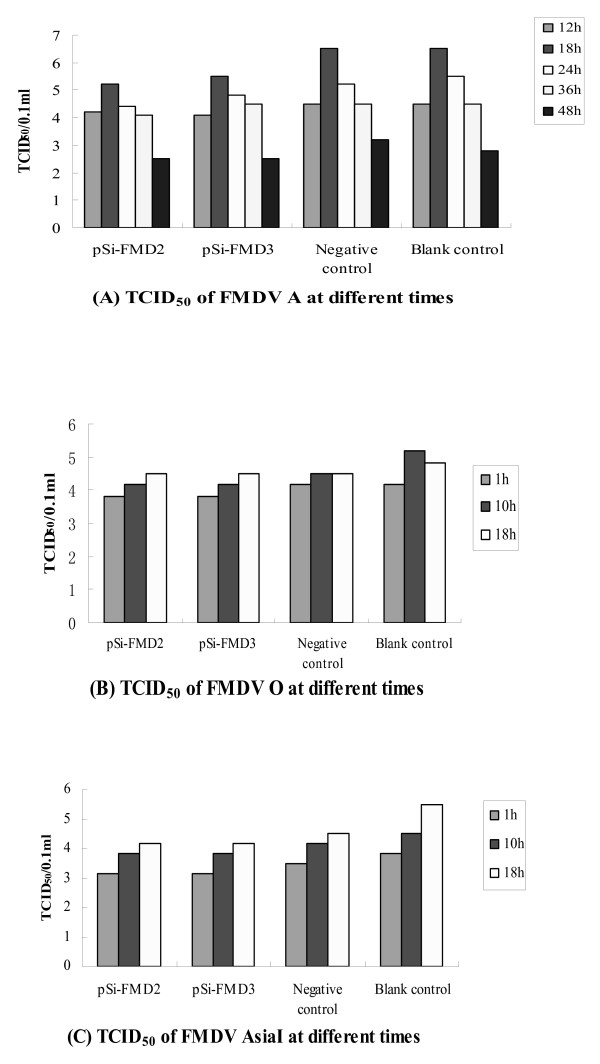
**TCID_50 _of the FMDV serotypes A, O, and Asia I at different times**. (A): TCID_50 _of FMDV A at different times. (B): TCID_50 _of FMDV O at different times. (C): TCID_50 _of FMDV AsiaI at different times. The TCID_50 _of the FMDV serotypes A, O, and Asia I detected in supernatants collected from cells transfected with FMDV-specific siRNA-expressing plasmids was lower than that in the control cells.

We determined first whether transient siRNAs expression could trigger an antiviral response on BHK-21 cell infected with FMDV. Transient cellular transfection and identification of FMDV were conducted in BHK-21 cells. Twenty four hours post-transfection, the transfected cells were infected with 5 × 10^3 ^TCID_50_/cell of FMDV serotypes A, O, and Asia1. The CPEs of the BHK-21 cells were observed at 10, 12, 18, 24, 36 and 48 h postinfection. Samples of supernatant were obtained at designated time points, and the TCID_50 _were determined by the Reed-Muench formula. BHK-21 cells are fibroblastic, growing in a monolayer, and having a well-defined tendency for parallel orientation. Viral infection causes a marked CPE resulting in total cellular detachment, rounding, and destruction, which can be observed under a microscope. As shown in Fig. 1, CPEs appeared in the BHK-21 cells infected with FMDV serotype A at 12 h postinfection and were particularly severe among the 4 groups between 24 h to 36 h. Cellular detachment, rounding, and destruction of the control group were more severe than the experimental group. At 48 h postinfection, the cells of the control group were dead and almost detached. CPEs appeared in the BHK-21 cells infected with FMDV serotype O and Asia I at 6–8 h postinfection and were particularly severe at 10–12 h postinfection. To further substantiate the antiviral activity, we determined the virus yield of cells infected with the 3 viruses at designated time points. The TCID_50 _of the FMDV serotypes A, O, and Asia I detected in supernatants collected from cells transfected with FMDV-specific siRNA-expressing plasmids was lower than that in the control cells.(Fig. 2) However, no significant inhibition was observed after 48 h (FMDV serotype A) and 18 h (FMDV serotypes O and Asia I). These results suggest that transient expression of FMDV hairpin RNA is competent to trigger an antiviral response on BHK-21 cell.

To further test the anti-FMDV activity of the siRNAs, we challenged Kunming White suckling mice (2–3 days old and weighing 3–4 g). The suckling mice were subcutaneously injected in the neck with 50–100 ug of plasmids dissolved in 100 ul of saline. Mice of the control group were subcutaneously injected with saline. After 6 h, the suckling mice were challenged with 5 and 20 LD_50 _of the FMDV serotypes  O, and Asia I per 0.1 milliliter by subcutaneous injection in the neck near the site that received the injected DNA and were then observed for 5–6 days postchallenge. All saline-injected mice (n _ 10 mice per group) died within 69 h, with most mice dying within 48 h, after the viral challenge. Only 3 of 9–10 mice pretreated with pSi-FMD2 and 4 of 10 mice pretreated with pSi-FMD3, survived a viral challenge of 5 LD_50 _for 5 days of observation. Further, only 1 of 9 mice pretreated with pSi-FMD2 and 1 of 9–10 mice pretreated with pSi-FMD3 survived a viral challenge of 20 LD_50 _for 5 days of observation. The percentage survival is shown in tables 1 and tables 2. Thus, table 1 and tables 2 clearly indicate that the mice treated with siRNA-expressing plasmids had reduced susceptibility to virus infection.

**Table 1:** The survival of mice challenged by FMDV AsiaI

	saline	FMD2	survival	FMD3	survival
5LD_50_	died within 69 h	3/9	33.3%	4/10	40%
20LD_50_	died within 69 h	1/9	11.1%	1/9	11.1%

The suckling mice were subcutaneously injected in the neck with 50–100 ug of plasmids dissolved in 100 ul of saline. After 6 h, the suckling mice were challenged with 5 LD_50 _and 20 LD_50 _FMDV serotypes Asia I per 0.1 milliliter by subcutaneous injection in the neck near the site that received the injected DNA and were then observed for 5–6 days postchallenge. All saline-injected mice (n _ 10 mice per group) died within 69 h, with most mice dying within 48 h, after the viral challenge. Only 3 of 9 mice pretreated with pSi-FMD2 and 4 of 10 mice pretreated with pSi-FMD3, survived a viral challenge of 5 LD_50 _for 5 days of observation. Further, only 1 of 9 mice pretreated with pSi-FMD2 and 1 of 9 mice pretreated with pSi-FMD3 survived a viral challenge of 20 LD_50 _for 5 days of observation.

**Table 2:** The survival of mice challenged by FMDV O

	saline	FMD2	survival	FMD3	survival
5LD_50_	died within 69 h	3/10	30%	4/10	40%
20LD_50_	died within 69 h	1/10	10%	1/10	10%

The suckling mice were subcutaneously injected in the neck with 50–100 ug of plasmids dissolved in 100 ul of saline. After 6 h, the suckling mice were challenged with 5 LD_50 _and 20 LD_50 _FMDV serotypes 0 per 0.1 milliliter by subcutaneous injection in the neck near the site that received the injected DNA and were then observed for 5–6 days postchallenge. All saline-injected mice (n _ 10 mice per group) died within 69 h, with most mice dying within 48 h, after the viral challenge. Only 3 of 10 mice pretreated with pSi-FMD2 and 4 of 10 mice pretreated with pSi-FMD3, survived a viral challenge of 5 LD_50 _for 5 days of observation. Further, only 1 of 10 mice pretreated with pSi-FMD2 and 1 of 10 mice pretreated with pSi-FMD3 survived a viral challenge of 20 LD_50 _for 5 days of observation.

In this work, it was demonstrated that transfection of BHK-21 cells with the 2 siRNA-expressing plasmids could induce a lower CPE compared with the controls. Further, the TCID_50 _of the FMDV serotypes A, O, and Asia I detected in supernatants collected from cells transfected with FMDV-specific siRNA-expressing plasmids was lower than that of control cells. On the other hand, expression of a 21-nt siRNA heterologous to the FMDV genome did not significantly reduce virus replication. In addition, when challenged by 5 LD_50 _or 20 LD_50 _of the FMDV serotypes O, or Asia I after injecting FMDV-specific siRNA-expressing plasmids, 10–40% suckling mice could resist virus infection. This report, as well as the results of others [22,23] suggests that double-stranded RNA (dsRNA) is a very powerful tool for the inhibition of virus replication and has a high therapeutic potential. In our case, the inhibition effect is not so well-defined as in the result reported by Chen et al [24] and Ronen Kahana et al [25]. However, in this study, siRNAs targeting 2 highly conserved sequences that could inhibit 3 viral serotypes were designed. Further research is required to determine whether this is the case for the other serotypes also.

## Competing interests

The authors declare that they have no competing interests.

## Authors' contributions

CCF, GZR Design and conception of study, WPY Plasmids constructs and inhibition analysis, WPY manuscript preparation. RY Breeding of mouse. All authors read and approved the final manuscript.

## References

[B1] Pereira HG, Gibbs EPJ (1981). Foot-and-mouth disease. Virus diseases of food animals.

[B2] Waterhouse PM, Wang MB, Lough T (2001). Gene silencing as an adaptive defense against viruses. Nature.

[B3] Zamore PD, Tuschl T, Sharp PA, Bartel DP (2000). RNAi: double-stranded RNA directs the ATP-dependent cleavage of mRNA at 21 to 23 nucleotide intervals. Cell.

[B4] Vance V, Vaucheret H (2001). RNA silencing in plants-defense and counter-defense. Science.

[B5] Gitlin L, Andino R (2003). Nucleic acid-based immune system: the antiviral potential of mammalian RNA silencing. J Virol.

[B6] Fire A (1999). RNA-triggered gene silencing. Trends Genet.

[B7] Leib DA, Machalek MA, Williams BR, Silverman RH, Virgin HW (2000). Specific phenotypic restoration of an attenuated virus by knockout of a host resistance gene. Proc Natl Acad Sci USA.

[B8] Stark GR, Kerr IM, Williams BR, Silverman RH, Schreiber RD (1998). How cells respond to interferons. Annu Rev Biochem.

[B9] Cullen BR (2002). RNA interference: antiviral defense and genetic tool. Nat Immunol.

[B10] Elbashir SM, Harborth J, Lendeckel W, Yalcin A, Weber K, Tuschl T (2001). Duplexes of 21-nucleotide RNAs mediate RNA interference in cultured mammalian cells. Nature.

[B11] Lee NS, Dohjima T, Bauer G, Li H, Li MJ, Ehsani A, Salvaterra P, Rossi J (2002). Expression of small interfering RNAs targeted against HIV-1 rev transcripts in human cells. Nat Biotechnol.

[B12] Novina CD, Murray MF, Dykxhoorn DM, Beresford PJ, Riess J, Lee SK, Collman RG, Lieberman J, Shankar P, Sharp PA (2002). siRNA-directed inhibition of HIV-1 infection. Nat Med.

[B13] Shlomai A, Shaul Y (2003). Inhibition of hepatitis B virus expression and replication by RNA interference. Hepatology.

[B14] Song E, Lee SK, Wang J, Ince N, Ouyang N, Min J, Chen J, Shankar P, Lieberman J (2003). RNA interference targeting Fas protects mice from fulminant hepatitis. Nat Med.

[B15] Gitlin L, Karelsky S, Andino R (2002). Short interfering RNA confers intracellular antiviral immunity in human cells. Nature.

[B16] Jiang M, Milner J (2002). Selective silencing of viral gene expression in HPV-positive human cervical carcinoma cells treated with siRNA, a primer of RNA interference. Oncogene.

[B17] Pfeffer S, Sewer A, Lagos-Quintana M, Sheridan R, Sander C, Grasser FA, van Dyk LF, Ho CK, Shuman S, Chier M, Russo JJ, Ju J, Randall G, Lindenbach BD, Rice CM, Simon V, Zavolan M, Tuschl T (2005). Identification of microRNAs of the herpesvirus family. Nat Methods.

[B18] Sullivan CS, Grundhoff AT, Tevethia S, Pipas JM, Ganem D (2005). SV40-encoded microRNAs regulate viral gene expression and reduce susceptibility to cytotoxic T cells. Nature.

[B19] Cullen BR (2005). RNAi the natural way. Nat Genet.

[B20] Katze MG, He Y, Gale M (2002). Viruses and interferon: a fight for supremacy. Nat Rev Immunol.

[B21] Stram Y, Molad T (1997). A ribozyme targeted to cleave the polymerase gene sequences of different foot-and-mouth disease virus (FMDV) serotypes. Virus Genes.

[B22] Park W-S, Miyano-Kurosaki N, Hayafune M, Nakajima E, Matsuzaki T, Shimada F, Takaku H (2002). Prevention of HIV-1 infection in human peripheral blood mononuclear cells by specific RNA interference. Nucleic Acids Res.

[B23] Capodici J, Kariko K, Weissman D (2002). Inhibition of HIV-1 infection by small interfering RNA-mediated RNA interference. J Immunol.

[B24] Chen W, Yan W, Du Q, Fei Li, Niu M, Ni Z, Sheng Z, Zheng Z (2004). RNA Interference Targeting VP1 Inhibits Foot-and-Mouth Disease Virus Replication in BHK-21 Cells and Suckling Mice. Journal of Virology.

[B25] Kahana Ronen, Kuznetzova Larisa, Rogel Arie (2004). Inhibition of foot-and-mouth disease virus replication by small interfering RNA. Journal of General Virology.

